# Vonoprazan-amoxicillin dual therapy vs. proton pump inhibitor based dual therapy for *Helicobacter pylori* eradication: a meta-analysis

**DOI:** 10.3389/fmed.2025.1739284

**Published:** 2026-01-14

**Authors:** You Lv, Hong-Hua Zhou, Jia-Geng Sun, Yu Qin, Ben-Gang Zhou

**Affiliations:** 1 Department of Gastroenterology, Yangzhou Dong Fang Hospital, Yangzhou, Jiangsu, China; 2 Department of Gastroenterology, Tianchang People's Hospital, Chuzhou, Anhui, China; 3 Department of Gastroenterology, Northern Jiangsu People's Hospital, Yangzhou, Jiangsu, China

**Keywords:** amoxicillin, dual therapy, *Helicobacter pylori*, meta-analysis, proton pump inhibitor, vonoprazan

## Abstract

**Background and objective:**

In recent years, the use of high-dose dual therapy based on PPI and amoxicillin (PPI-HDDT) has shown satisfactory efficacy in eradicating *Helicobacter pylori* (*H. pylori*) as a first-line treatment. Vonoprazan (VPZ), a new potassium-competitive acid blocker, exhibits rapid, potent, and enduring acid inhibitory effects in comparison to PPI. However, to date, evidence comparing VPZ-based HDDT (VPZ-HDDT) with PPI-HDDT for eradication of *H. pylori* remains limited. This meta-analysis aimed to systematically evaluate the effect of the two regimens for first-line *H. pylori* eradication.

**Methods:**

This meta-analysis searched PubMed, Embase, Cochrane Library, and Web of Science (from inception to October 1, 2025) for randomized controlled trials (RCTs) comparing VPZ-HDDT vs. PPI-HDDT in treatment-naive *H. pylori* patients. Utilize a random-effects model for meta-analysis to ascertain the pooled relative risk (RR) with a 95% confidence interval (CI).

**Results:**

Four RCTs (involving 1,807 patients) were included. Based on the intention-to-treat (ITT) and per-protocol (PP) analysis data, the results of the meta-analysis consistently demonstrated that the *H. pylori* eradication rate in the VPZ-HDDT group is higher than that in the PPI-HDDT group (ITT analysis: 88.0 vs. 82.7%, RR = 1.05, 95% CI:1.00–1.10, *P* = 0.04; *I*^2^ = 28%; PP analysis: 92.3 vs. 87.3%, RR = 1.04, 95% CI:1.01–1.08, *P* = 0.02; *I*^2^ = 15%). There were no statistically significant differences in the incidence of total adverse events (8.6 vs. 10.6%, RR = 0.78, 95% CI:0.59–1.04, *P* = 0.09) and compliance (97.1 vs. 95.6%, RR = 1.02, 95% CI:0.99–1.05, *P* = 0.22) between the VPZ-HDDT group and the PPI-HDDT group.

**Conclusions:**

VPZ-HDDT exhibits superior *H. pylori* eradication rate over the PPI-HDDT regimen, with comparable safety and compliance.

**SystematicReviewRegistration:**

https://osf.io/fxju2.

## 1 Introduction

*Helicobacter pylori* (*H. pylori*), a highly prevalent chronic pathogenic bacterium worldwide, is closely associated with the development and progression of various digestive system diseases, including chronic gastritis, peptic ulcers, and gastric cancer (1, 2). These conditions pose a significant threat to human digestive health and quality of life. Currently, the eradication of *H. pylori* has become a key intervention for the prevention and treatment of the aforementioned diseases.

Eradication regimens commonly used consist of triple therapy (proton pump inhibitor plus two different antibiotics) and bismuth quadruple therapy (BQT, bismuth plus triple therapy) (2, 3). However, the escalating antibiotic resistance, notably to clarithromycin, has rendered successful eradication increasingly challenging, markedly diminishing the efficacy of the conventional triple therapy (4). Despite BQT yielding relatively satisfactory eradication rates, challenges such as regimen complexity, elevated occurrence of adverse reactions, and increased costs persist (5, 6), underscoring the need for an effective, safe, and economically viable novel eradication regimen.

In recent years, high-dose dual therapy (HDDT) with proton pump inhibitor (PPI) and amoxicillin (3,000 mg/day) has received widespread attention from gastroenterologists. Prior research has demonstrated that PPI-based HDDT (PPI-HDDT) can achieve similar eradication rates and compliance as BQT, but with a lower incidence of adverse reactions (5.9 vs. 34.1%) (7). In the HDDT regimen, increasing the total dose and dosing frequency of PPI and amoxicillin (3 or 4 times daily) is more advantageous for maintaining stable PPI blood concentrations and creating a stable high pH environment in the stomach. This aids in promoting the proliferation of *H. pylori* and enhancing its sensitivity to amoxicillin (5–7).

Adequate acid suppression is crucial for the successful eradication of *H. pylori* (8). Given the significance of acid suppression, novel acid-suppressing agents have been investigated to enhance eradication efficacy. Vonoprazan (VPZ), a novel potassium-competitive acid blocker (P-CAB), has been widely used in acid-related diseases (9–11). Recent studies have shown that VPZ-amoxicillin dual therapy outperforms PPI-based triple therapy or BQT in eradicating *H. pylori*, underscoring the benefits of robust acid suppression in enhancing treatment efficacy (12, 13). However, to date, evidence comparing VPZ-based HDDT (VPZ-HDDT) with PPI-HDDT for first-line eradication of *H. pylori* remains limited. At present, there is no meta-analysis available that specifically compares VPZ-HDDT and PPI-HDDT for first-line *H. pylori* eradication.

Therefore, this study aims to address this research gap by systematically searching for randomized controlled trials (RCTs) comparing the two regimens for first-line *H. pylori* eradication to determine whether VPZ-HDDT offers potential advantages as a first-line treatment option for eradicating *H. pylori*.

## 2 Materials and methods

### 2.1 Research registration and reporting standards

This study has been pre-registered on the Open Science Framework platform (https://osf.io/fxju2) and followed the PRISMA standards for reporting (14).

### 2.2 Data sources and search strategies

Searches were performed on the PubMed, Embase, the Cochrane Library, and the Web of Science databases, spanning from their establishment to October 1, 2025. The main search formula was as follows: (“*Helicobacter*” OR “*Helicobacter pylori*” OR “*Campylobacter pylori*” OR “*H. pylori*”) AND (“vonoprazan” OR “TAK-438” OR “TAK438” OR “takecab” OR “potassium-competitive acid blockers” OR “potassium-competitive acid blocker”) AND (“amoxicillin” OR “amoxycillin”) AND (“dual therapy” OR “high-dose dual therapy”). We employed a combination of MeSH terms and free text in our search, without any language restrictions. We adjust the search strategies based on the characteristics of different databases.

### 2.3 Inclusion and exclusion criteria

Inclusion criteria: (1) The study design must be parallel-group RCTs that has been published; (2) Participants should be patients with initial *H. pylori* infection undergoing treatment for the first time; (3) The study should involve a minimum of two groups, with one group receiving VPZ-HDDT and another receiving PPI-HDDT, amoxicillin should be administered at a daily dosage of 3,000 mg, either three times daily or four times daily; (4) The outcome measures should include the *H. pylori* eradication rate, adverse events, and compliance, with the *H. pylori* eradication rate being the primary outcome measure and the others being secondary outcome measures.

Exclusion criteria included: (1) duplicate publications; (2) non-randomized controlled studies; (3) trial protocols, trial registration records, conference abstracts, reviews, and meta-analyses.

### 2.4 Study selection, data extraction and risk of bias

Utilize Endnote X21 to automatically or manually remove duplicate studies and conduct literature screening based on inclusion and exclusion criteria. Two researchers independently screen literature, with any discrepancies resolved through consultation with a third researcher. Two researchers independently extracted data using a pre-designed data extraction form. The extracted key information included the first author's surname, publication year, study design, study country or region, participant age, sample size, specific details of the eradication regimen, diagnostic method for confirming *H. pylori* infection pre- and post-eradication, specific details of relevant outcome data (eradication rate, adverse events, and compliance). In cases of disagreement, an independent assessor was responsible for evaluating inconsistencies, followed by participation in deliberations until consensus was reached. Two researchers employed the latest revised version of the Cochrane Risk of Bias tool (RoB 2) for evaluating risk of bias (15). Any discrepancies were resolved through discussion, and a third researcher may be consulted if necessary.

### 2.5 Statistical analysis

Meta-analysis was conducted using Review Manager software 5.3 (The Cochrane Collaboration, Copenhagen, Denmark). A random-effects model was utilized to account for potential heterogeneity among studies. Intention-to-treat (ITT) and per-protocol (PP) analyses were employed to assess the *H. pylori* eradication rate. The effect measure was expressed as risk ratio (RR) with its corresponding 95% confidence interval (CI). Heterogeneity among trial results was assessed using Cochran's *Q* test (significance level set at *P* < 0.10) and *I*^2^ statistic (0%−25%: insignificant heterogeneity, 26%−50%: low heterogeneity, 51%−75%: moderate heterogeneity, ≥76%: high heterogeneity) (16). Subgroup analyses were conducted to explore any factors potentially influencing the overall results. Publication bias was evaluated using funnel plot and Egger's test if the meta-analysis included at least 10 studies (17, 18). Statistical significance was defined as a *P*-value <0.05.

## 3 Results

### 3.1 Search results and study selection process

**Figure 1:**
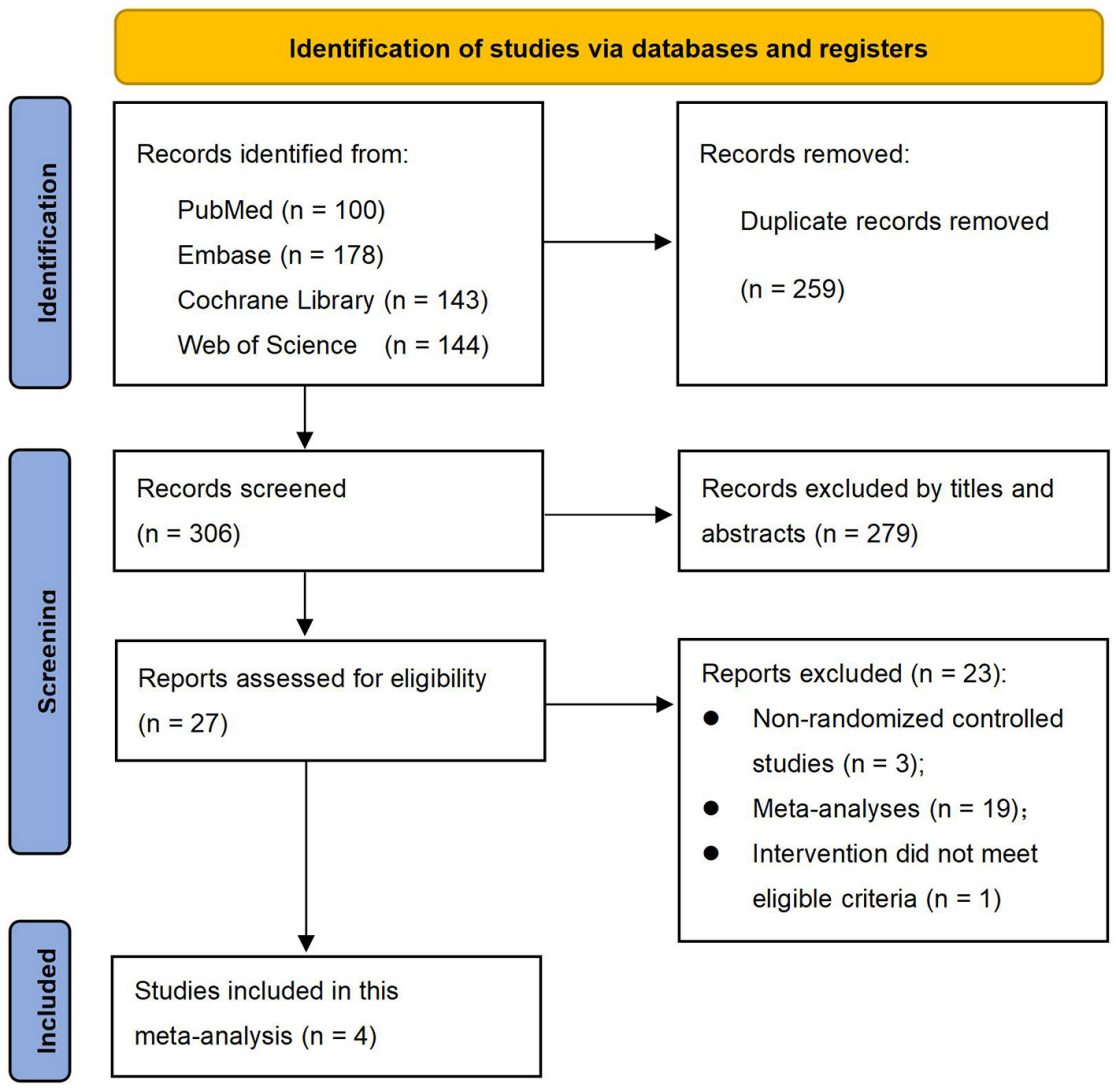
PRISMA flow diagram of literature retrieval and screening.

Initially, a total of 565 records were retrieved from four English electronic databases: PubMed (*n* = 100), Embase (*n* = 178), the Cochrane Library (*n* = 143), and Web of Science (*n* = 144). Following the elimination of 259 duplicate records, a further 279 records were excluded through title and abstract screening, resulting in 34 studies necessitating full-text review. Subsequently, during the full-text evaluation phase, three studies were non-randomized controlled studies, nineteen were meta-analyses, and one study's intervention did not meet the inclusion criteria. These studies (*n* = 23) were all subsequently excluded. Ultimately, 4 RCTs (19–22) meeting the criteria were selected for inclusion in the meta-analysis. The flowchart depicted in Figure 1 outlines the study selection process.

### 3.2 Study characteristics

The publication language of the four included RCTs was English, with publication dates ranging from 2023 to 2025. All studies were conducted in China, with three being multicenter RCTs (19, 21, 22) and one being a single-center RCT (20). Among these studies, one was a three-arm trial (20), while the remaining three were two-arm trials (19, 21, 22). The total sample size ranged from 121 to 690. Regarding the VPZ-HDDT regimen, two RCTs (20, 22) had a duration of 14 days, while three RCTs (19–21) had a duration of 10 days. The amoxicillin dosage in the 14-day VPZ-HDDT regimen was 750 mg per dose four times daily, however, in the 10-day VPZ-HDDT regimen, it was 1,000 mg per dose three times daily. For the PPI-HDDT dual regimen, the duration was 14 days in all trials, with one (19) using rabeprazole as the PPI and three using esomeprazole (20–22). The initial diagnostic methods for *H. pylori* included the urea breath test (UBT), rapid urease test (RUT), histology, or bacterial culture, while post-treatment reexamination typically involved UBT. Table 1 summarizes the basic characteristics of the four RCTs.

**Table 1:** Characteristics of included studies.

**Author (year)**	**Study design**	**No. of patients (VPZ-HDDT/PPI-HDDT)**	**Mean age (VPZ-HDDT/PPI-HDDT)**	**VPZ-HDDT dual regimen**	**PPI-HDDT dual regimen**	**Diagnositic methods of** ***H. pylori***	
						**Initial**	**Reexamination**
Han (2023) (19)	Multicenter RCT	690 (345/345)	42.5/41.8	Vonoprazan 20 mg BID, amoxicillin 1,000 mg TID, 10 days	Rabeprazole 10 mg TID, amoxicillin 1,000 mg TID, 14 days	^13^C/^14^C-UBT/histology	^13^C/^14^C-UBT
Yan (2024) (20)	Single-center RCT	426 (142/142/142)	48.2/47.9/47.2	Group-I: vonoprazan 20 mg BID, amoxicillin 750 mg QID, 14 days group-II: vonoprazan 20 mg BID, amoxicillin 1,000 mg TID, 10 days	Esomeprazole 20 mg QID, amoxicillin 1,000 mg TID, 14 days	^13^C/^14^C-UBT	^13^C-UBT
Zhou (2024) (21)	Multicenter RCT	570 (287/283)	46.9/47.7	Vonoprazan 20 mg BID, amoxicillin 1,000 mg TID, 10 days	Esomeprazole 20 mg QID, amoxicillin 750 mg QID, 14 days	^13^C/^14^C-UBT	^13^C/^14^C-UBT
Tai (2025) (22)	Multicenter RCT	121 (61/60)	56.5/53.9	Vonoprazan 20 mg BID, amoxicillin 750 mg QID, 14 days	Esomeprazole 40 mg TID, amoxicillin 750 mg QID, 14 days	RUT/histology/culture/^13^C-UBT	^13^C-UBT

VPZ, vonoprazan; PPI, proton pump inhibitor; HDDT, high-dose dual therapy; RCT, randomized controlled trial; BID, twice daily; TID, three times daily; QID, four times daily; UBT, urea breath test; RUT, rapid urease test.

### 3.3 Risk of bias assessment

For primary outcome (*H. pylori* eradication rate), all four RCTs demonstrated low risk of bias in terms of missing outcome data, measurement of the outcome, and selection of the reported result. Three RCTs (19, 21, 22) had low risk of bias in the randomization process, while one RCT (20) was judged to have some concerns due to unclear randomization procedures. Detailed information on bias risk is provided in Supplementary Figures S1, S2 and Supplementary Table S1.

### 3.4 *H. pylori* eradication rate

Data regarding the *H. pylori* eradication rate based on ITT and PP analyses was provided in all four included trials.

**Figure 2:**
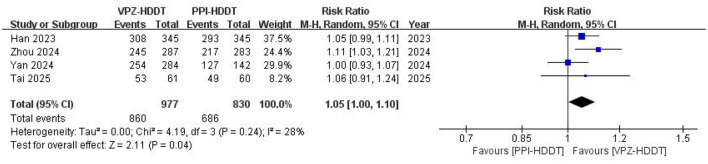
Forest plot of the *H. pylori* eradication rate (ITT analysis).

**Figure 3:**
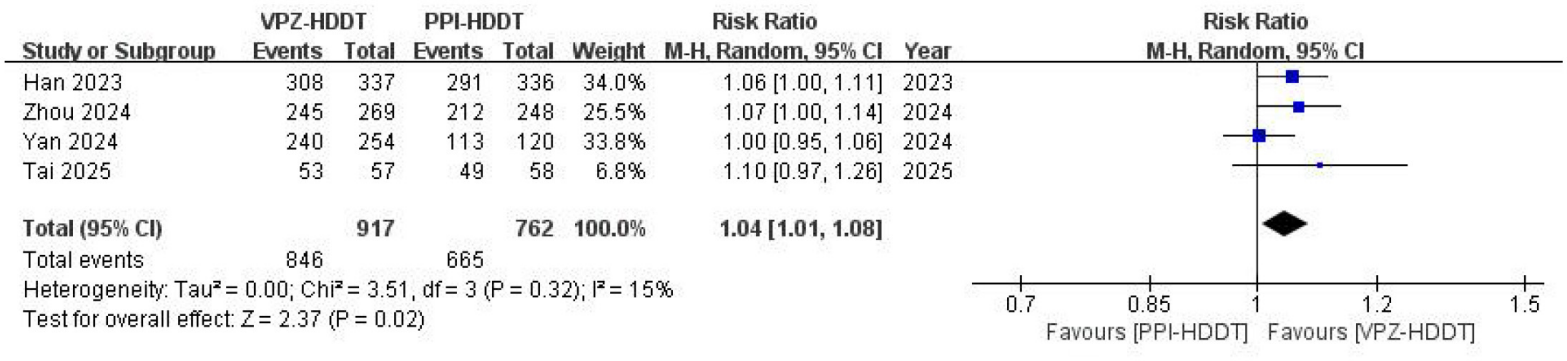
Forest plot of the *H. pylori* eradication rate (PP analysis).

Based on the ITT analysis data, the meta-analysis demonstrated a statistically significant higher eradication rate of VPZ-HDDT compared to PPI-HDDT (88.0 vs. 82.7%, RR = 1.05, 95% CI: 1.00–1.10, *P* = 0.04; Figure 2). Low heterogeneity was observed (*I*^2^ = 28%, *P* = 0.24). The similar conclusion was also obtained in the PP analysis (92.3 vs. 87.3%, RR = 1.04, 95% CI: 1.01–1.08, *P* = 0.02), with insignificant heterogeneity (*I*^2^ = 15%, *P* = 0.32; Figure 3).

**Figure 4:**
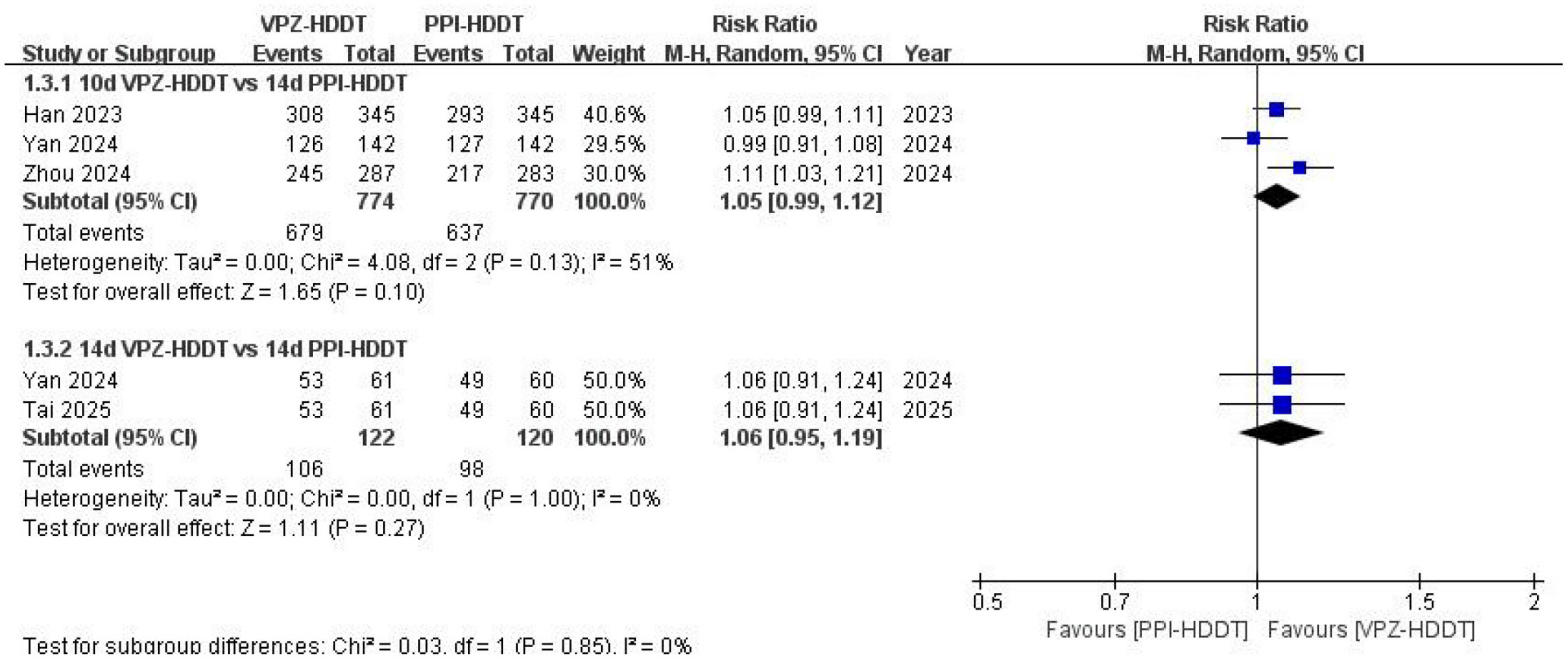
Forest plot for subgroup analysis based on the duration of VPZ-HDDT on *H. pylori* eradication rate.

**Figure 5:**
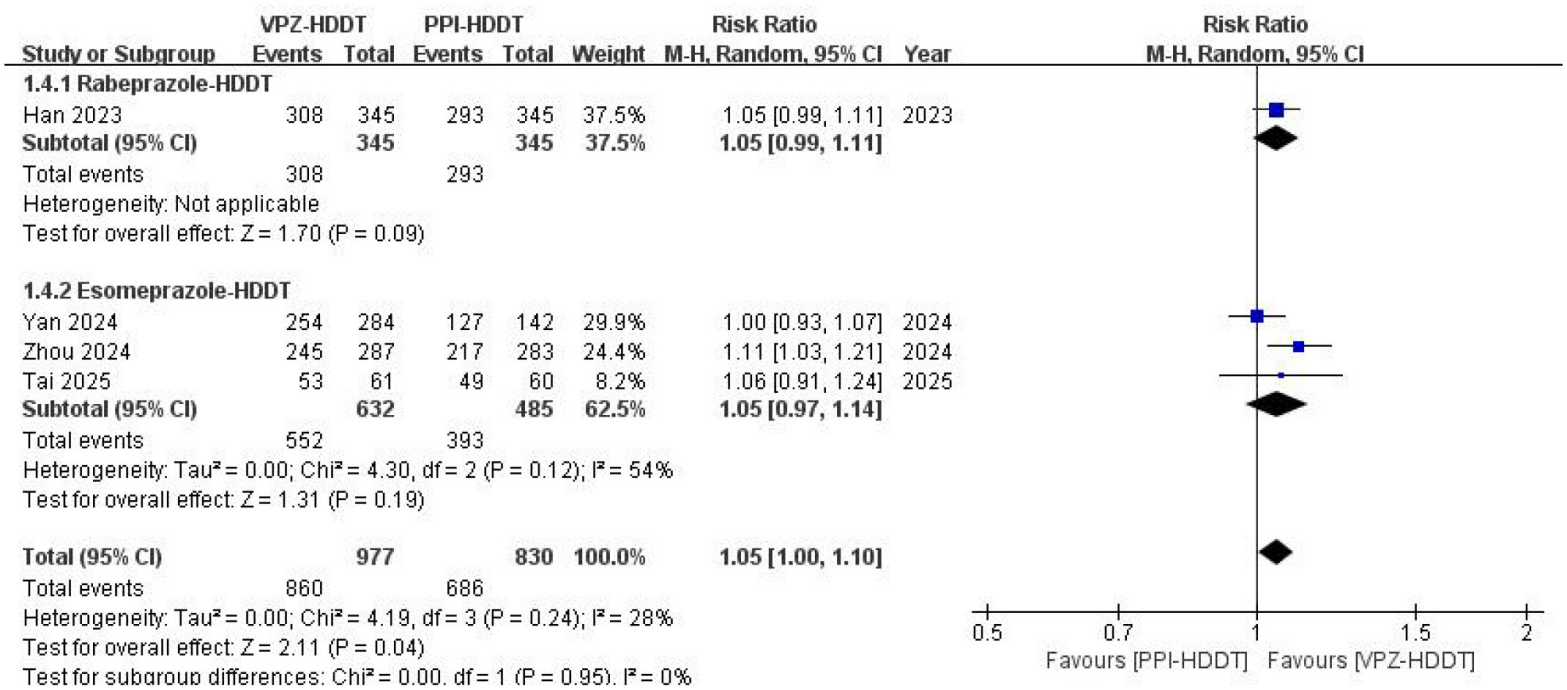
Forest plot for subgroup analysis based on different types of PPIs on *H. pylori* eradication rate.

Based on the data from the ITT analysis, we further conducted a subgroup analysis according to the treatment duration of the VPZ-HDDT regimen and different types of PPIs to explore potential factors affecting the overall results. The results of the subgroup analysis based on the treatment duration of the VPZ-HDDT regimen (10 and 14 days) showed that in the 10-day subgroup (*n* = 3 RCTs, 87.7 vs. 82.7%, RR = 1.05, 95% CI: 0.99–1.12, *P* = 0.10) and the 14-day subgroup (*n* = 2 RCTs, 86.9 vs. 81.7%, RR = 1.06, 95% CI: 0.95–1.19, *P* = 0.27), the eradication rate of the VPZ-HDDT group was higher than that of the PPI-HDDT group; however, the differences were not statistically significant (Figure 4). Similarly, in subgroup analyses based on different types of PPIs, no significant statistical differences in terms of eradication rate were found between the two groups, whether using rabeprazole-HDDT (*n* = 1 RCT, 89.3 vs. 84.9%, RR = 1.05, 95% CI: 0.99–1.11, *P* = 0.09) or esomeprazole-HDDT (*n* = 3 RCTs, 87.3 vs. 81.7%, RR = 1.05, 95% CI: 0.97–1.14, *P* = 0.19; Figure 5).

### 3.5 Adverse events

#### 3.5.1 Total adverse events

**Figure 6:**
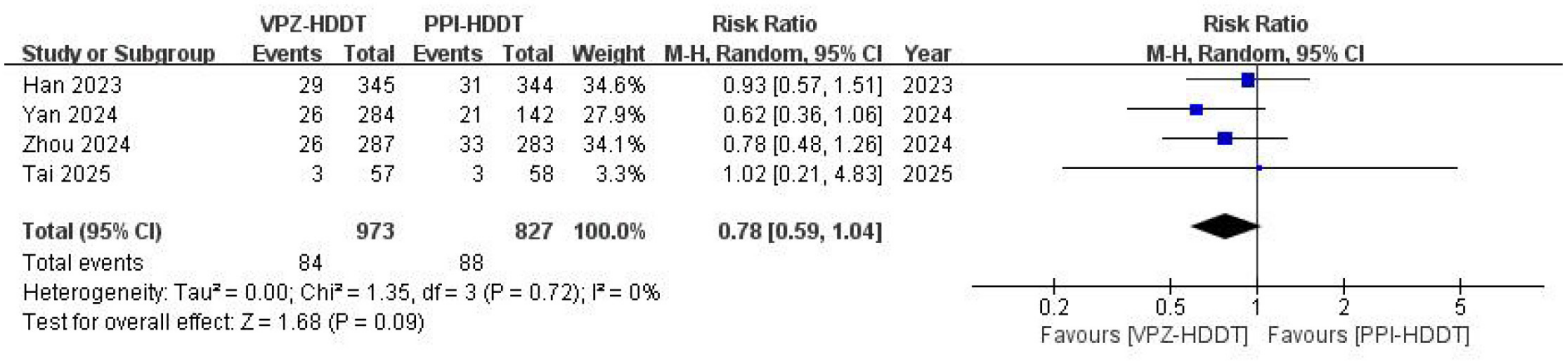
Forest plot of the incidence of total adverse events.

Four RCTs assessed the incidence rate of total adverse events. In the meta-analysis, it was found that the total adverse event rate of VPZ-HDDT was lower than that of PPI-HDDT, but the difference was not statistically significant (8.6 vs. 10.6%, RR = 0.78, 95% CI: 0.59–1.04, *P* = 0.09). There was no heterogeneity observed (*I*^2^ = 0, *P* = 0.72; Figure 6).

#### 3.5.2 Specific adverse events

**Figure 7:**
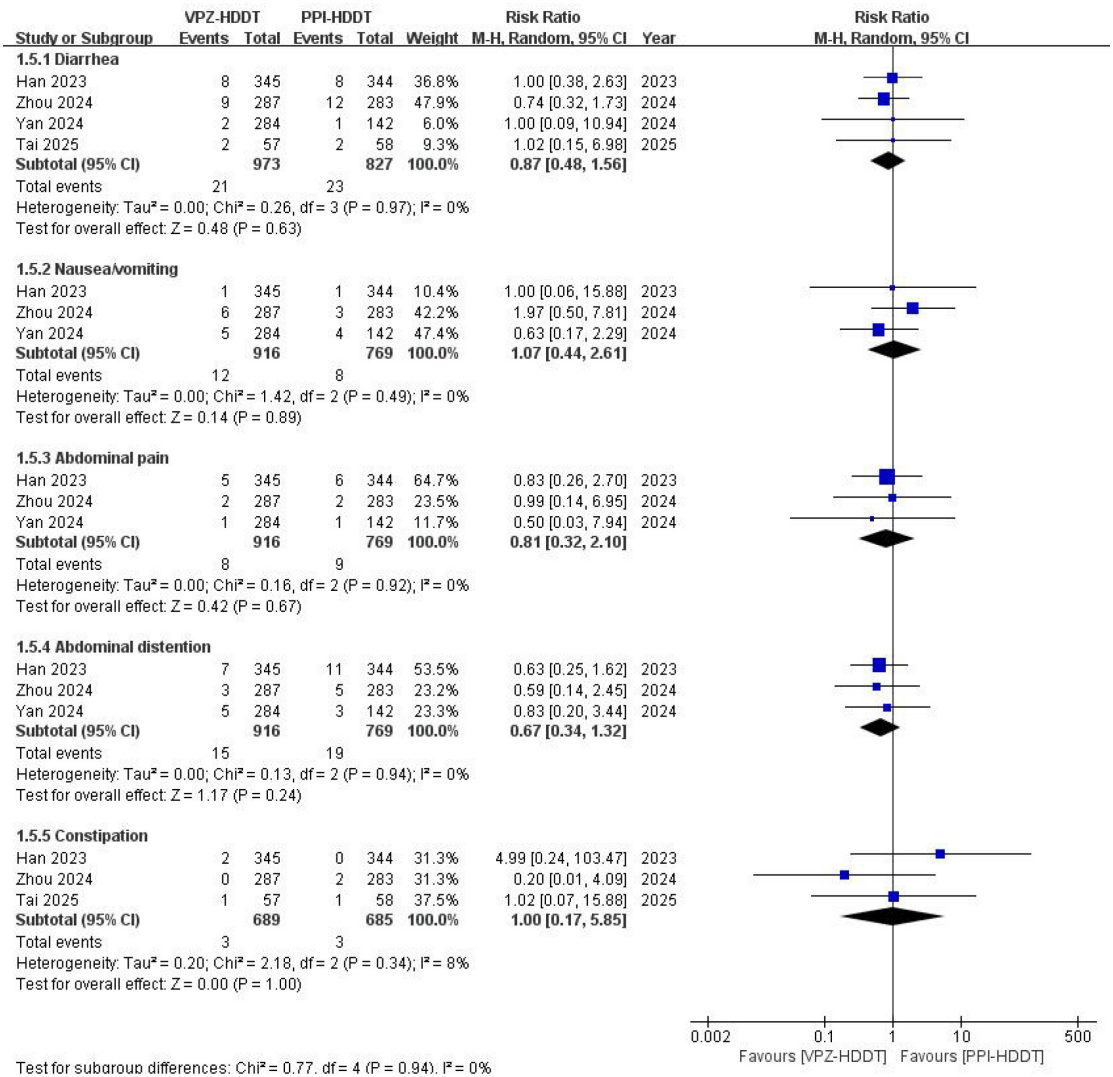
Forest plot of the specific gastrointestinal adverse events.

**Figure 8:**
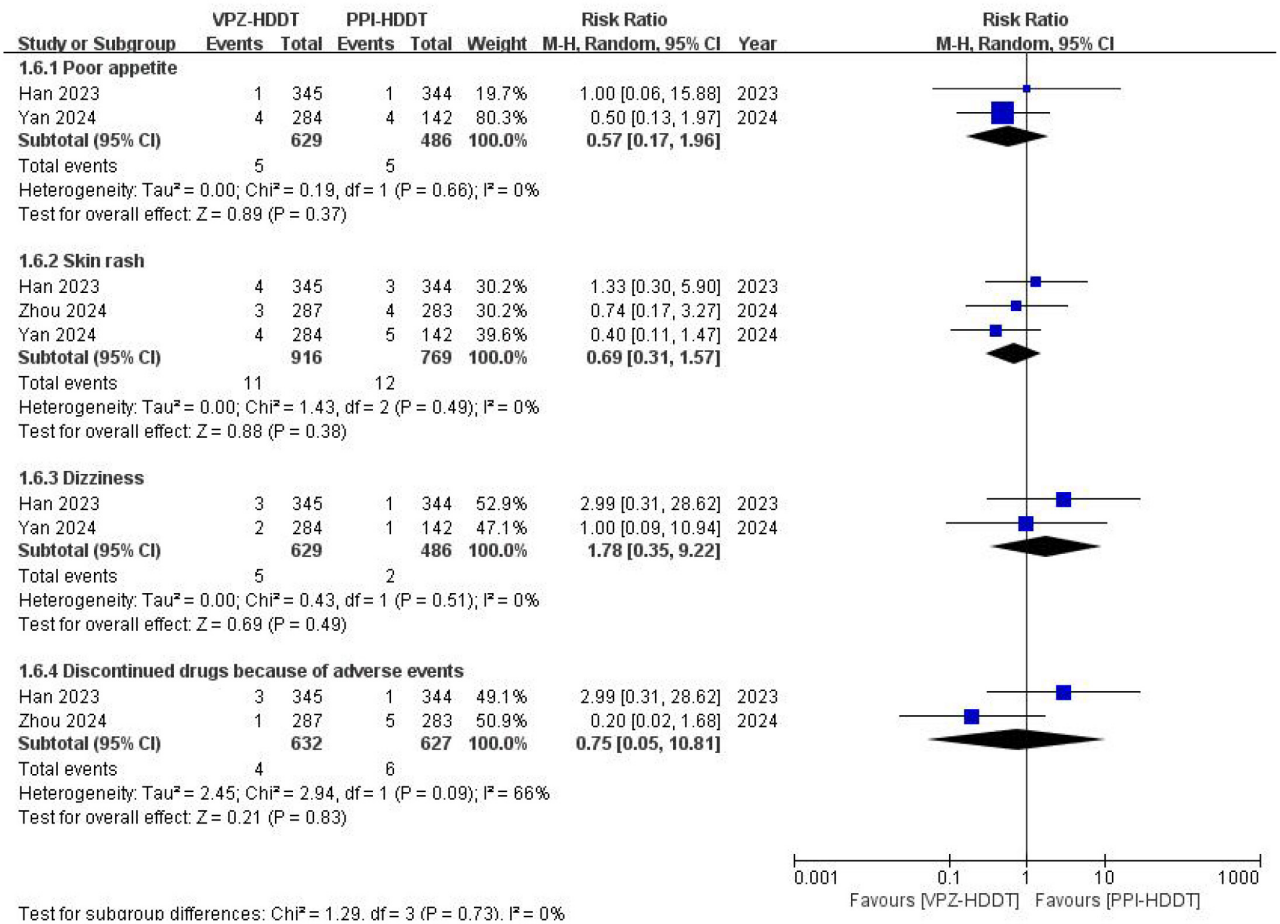
Forest plot of the other specific adverse events.

Regarding the incidence of specific gastrointestinal adverse events, all trials reported the occurrence of diarrhea, with three trials (19–21) also reporting rates of nausea/vomiting, abdominal pain, and bloating, while three trials (19, 21, 22) reported rates of constipation. The results of the meta-analyses indicated that there were no statistically significant differences in the incidence rates of diarrhea (RR = 0.87, 95% CI: 0.48–1.56, *P* = 0.63), nausea/vomiting (RR = 1.07, 95% CI: 0.44–2.61, *P* = 0.89), abdominal pain (RR = 0.81, 95% CI: 0.32–2.10, *P* = 0.67), abdominal distention (RR = 0.67, 95% CI: 0.34–1.32, *P* = 0.24), and constipation (RR = 1.00, 95% CI: 0.17–5.85, *P* = 1.00) between the VPZ-HDDT group and the PPI-HDDT group (Figure 7). Similarly, there were no statistically significant differences between the two groups in specific adverse reactions such as poor appetite, skin rash, dizziness, and discontinued drugs due to adverse events (Figure 8). Only two RCTs (19, 21) reported severe adverse events, with four cases each in the VPZ-HDDT and PPI-HDDT groups, primarily consisting of rash, diarrhea, and abdominal pain. The results indicated no statistically significant difference in severe adverse events between the VPZ-HDDT and PPI-HDDT groups.

### 3.6 Compliance

**Figure 9:**
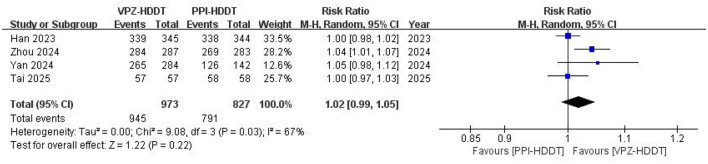
Forest plot of compliance.

All four RCTs reported compliance. The results of this meta-analysis indicated no significant difference in compliance between the VPZ-HDDT and PPI-HDDT groups (97.1 vs. 95.6%, RR = 1.02, 95% CI: 0.99–1.5, *P* = 0.22). Moderate heterogeneity was observed among the studies (*I*^2^ = 67%, *P* = 0.03; Figure 9).

## 4 Discussion

This meta-analysis systematically evaluated the efficacy and safety of VPZ-HDDT vs. PPI- HDDT for first-line *H. pylori* eradication, using data from 4 RCTs involving 1,807 patients. The core finding of this study revealed a significantly higher eradication rate of VPZ-HDDT compared to PPI-HDDT in both ITT (88.0 vs. 82.7%, *P* = 0.04) and PP analyses (92.3 vs. 87.3%, *P* = 0.02). This aligns with the unique pharmacological advantages of VPZ, emphasizing its potential in addressing unmet needs in the management of *H. pylori*.

VPZ, the first-in-class P-CAB, was first approved in Japan in 2014 as part of a combination regimen for treating *H. pylori* infection (23). It exerts its acid-inhibitory effect by directly blocking the active site of the H+/K+-ATPase in gastric parietal cells (23, 24). Unlike PPIs, VPZ does not require activation in an acidic environment and is not significantly influenced by CYP2C19 genetic polymorphism, leading to faster, more sustained, and effective acid suppression across different patient populations (9, 10, 25). This is crucial for optimizing the efficacy of amoxicillin, as the antibiotic's bactericidal activity against *H. pylori* is pH-dependent (optimal pH >6) (5–7). In HDDT regimens, maintaining stable high pH is further enhanced by frequent dosing of acid suppressants and amoxicillin (3 or 4 times daily); VPZ's superiority in acid control likely explains why VPZ-HDDT outperforms PPI-HDDT, even though both regimens use the same 3,000 mg/day dose of amoxicillin.

Notably, subgroup analyses by VPZ-HDDT duration (10 vs. 14 days) revealed numerically higher eradication rates in VPZ-HDDT but no statistical significance (*P* = 0.10 and *P* = 0.27, respectively). This lack of significance may stem from small sample sizes in subgroups or inherent variability in study populations. However, the consistency in trend across durations suggested that the efficacy of VPZ-HDDT was not strictly dependent on extended treatment - an important consideration for patient compliance. Indeed, compliance was comparable between the groups (97.1 vs. 95.6%, *P* = 0.22), reflecting that both the 10- and 14-day VPZ-HDDTs were well-tolerated in terms of regimen complexity. Recently, two multicenter RCTs (26, 27) have also shown similar eradication rates between the 10- and 14-day VPZ-HDDT regimens, indicating that both durations can achieve favorable eradication outcomes. In the included studies, different types of PPIs (rabeprazole or esomeprazole) were used in the control group for the PPI-HDDT regimen. Given the potential impact of different PPIs on outcomes, subgroup analyses were conducted based on the type of PPIs used. The results indicated that there was no statistically significant difference in eradication rates between VPZ-HDDT and rabeprazole-HDDT or esomeprazole-HDDT. The lack of statistical significance in the findings could be due to the small number of studies and samples within the subgroups.

Adverse events are commonly observed during the process of *H. pylori* eradication (28). In this meta-analysis, the overall incidence of adverse events in the VPZ-HDDT group (8.6%) was lower than in the PPI-HDDT group (10.6%); however, the difference between the two groups was not statistically significant. This aligns with the favorable safety profile of VPZ, in contrast to BQT, which exhibited a high adverse event rate of 34.1% (7). Similarly, there were no statistically significant differences between the two groups in specific gastrointestinal adverse events (such as diarrhea, nausea, and abdominal pain) or other events (such as rash and dizziness). The absence of increased adverse events with VPZ-HDDT is critical, as tolerability directly impacts compliance and real-world efficacy, especially in first-line settings where patient trust in treatment is foundational. Safety data further supports the clinical utility of the VPZ-HDDT regimen.

This study has strengths that enhance the reliability of its findings. First, it adheres to PRISMA guidelines and pre-registration, minimizing reporting bias. Second, rigorous study selection (only parallel-group RCTs, excluding non-randomized studies) and independent data extraction/bias assessment (using new RoB 2) reduce methodological heterogeneity. Most included RCTs were multicenter (3/4) and recent (2023–2025), reflecting contemporary clinical practice and increasing the relevance of results to current *H. pylori* management.

However, despite these strengths, several limitations must also be acknowledged. First, all RCTs were conducted in China, limiting generalizability to other regions with different *H. pylori* strains, antibiotic resistance patterns, or genetic backgrounds. Future research should address these gaps by conducting large-scale, multicenter RCTs in diverse geographic regions (e.g., Europe, North America) to validate the efficacy of VPZ-HDDT in varied populations. Second, the small number of included studies (*n* = 4) may limit the power to detect subtle differences in the incidence of adverse events or subgroup efficacy. This may affect the robustness and reliability of the results, necessitating further expansion of the study size and sample size to validate the aforementioned findings in greater depth in the future. Third, our meta-analysis showed low heterogeneity concerning the primary outcome, which may stem from variations in treatment regimens, amoxicillin dosing frequencies, and administration methods. Despite conducting thorough subgroup some analyses, limitations in study numbers and sample sizes make it challenging to detect subtle differences that could potentially impact the results. Finally, all included studies were conducted as open-label trials (non-blinded), which, although had no impact on the *H. pylori* eradication rate (an objective outcome), could have influenced the reporting of adverse events and medication compliance.

Given the global challenge of antibiotic resistance and the limitations of conventional regimens, our meta-analysis address an urgent clinical gap in *H. pylori* management: the need for effective, convenient, and well-tolerated first-line treatment options in the context of increasing antibiotic resistance and poor patient compliance. Firstly, compared to PPI-HDDT, VPZ-HDDT offers a more efficient treatment option, particularly for patients with poor response to conventional therapies or at risk of resistance. Additionally, in the PPI-HDDT regimen, PPIs need to be taken three or four times daily before meals, whereas in the VPZ-HDDT regimen, VPZ is administered only twice daily and is not affected by food intake. This regimen makes VPZ-HDDT more convenient than PPI-HDDT, with the advantage of easier administration likely to encourage patients to adhere to the treatment regimen in terms of timing and dosage throughout long-term therapy, thereby ensuring treatment efficacy.

## 5 Conclusion

In conclusion, this meta-analysis demonstrated that VPZ-HDDT achieved a superior first-line *H. pylori* eradication rate compared to PPI-HDDT, with comparable compliance and safety. Given the global challenge of antibiotic resistance and the limitations of conventional regimens, VPZ-HDDT represents a promising alternative for first-line *H. pylori* eradication, particularly in settings where sustained acid control and tolerability are priorities.

## Data Availability

The original contributions presented in the study are included in the article/Supplementary material, further inquiries can be directed to the corresponding author.
