# Supplementary material for: Vonoprazan-amoxicillin dual therapy vs. proton pump inhibitor based dual therapy for Helicobacter pylori eradication: a meta-analysis
Source: Front Med (Lausanne). 2026 Jan 14;12:1739284. doi: 10.3389/fmed.2025.1739284 (PMC12847323; doi:10.3389/fmed.2025.1739284)
Supplement: Supplementary file 1 [file Data_Sheet_1.pdf]

Supplementary online materials

Figure S1. **Risk of bias graph**

Figure S2. **Risk of bias summary**

Table S1. **Assessment of risk of bias of included studies**

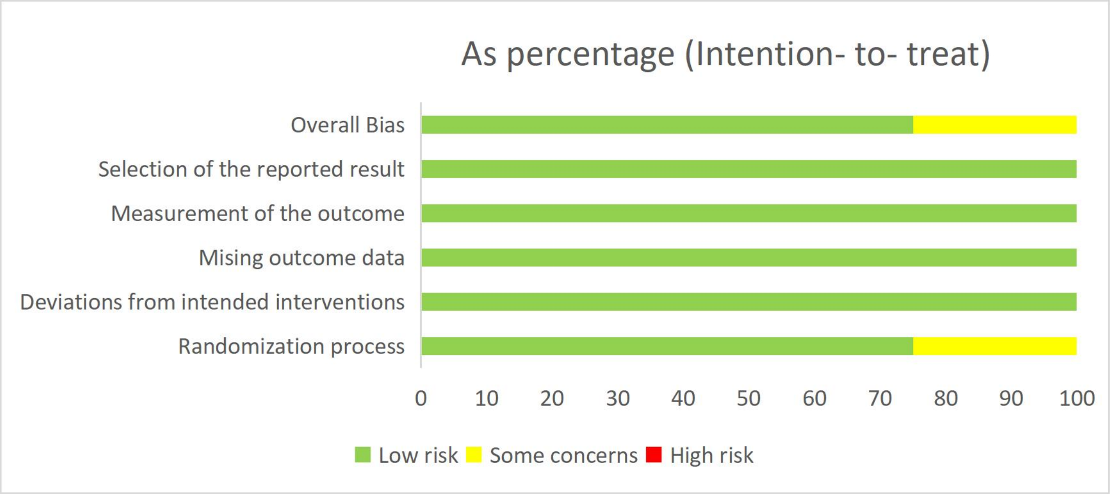

Figure S1. Risk of bias graph (*H. pylori* eradication rate)

Study ID	D1	D2	D3	D4	D5	Overall
Han 2023	+	+	+	+	+	+
Yan 2024	!	+	+	+	+	!
Zhou 2024	+	+	+	+	+	+
Tai 2025	+	+	+	+	+	+

D1 Randomisation process

D2 Deviations from the intended interventions

D3 Missing outcome data

D4 Measurement of the outcome

D5 Selection of the reported result

+ Low risk

! Some concerns

Figure S2. Risk of bias summary (*H. pylori* eradication rate)

Table S1 Assessment of risk of bias of included studies						
**First author and year**	**Randomisation process**	**Deviations from the intended interventions**	**Missing outcome data**	**Measurement of the outcome**	**Selection of the reported result**	**Overall assessment**
Han 2023	Computer-generated randomization, Centralization distribution (**low risk**)	**Open label trials**, deviations from established intervention measures do not affect objective outcome (*H. pylori* eradication rate), but may affect subjective outcomes (adverse events and compliance)	Although there were some missing data, the study conducted both ITT and PP analyses, and there is evidence that the result was not biased by missing outcome data (**low risk**)	Low risk	Low risk	Low risk
Yan 2024	Unclear (**Some concerns**)	**Open label trials**, deviations from established intervention measures do not affect objective outcome (*H. pylori* eradication rate), but may affect subjective outcomes (adverse events and compliance)	Although there were some missing data, the study conducted both ITT and PP analyses, and there is evidence that the result was not biased by missing outcome data (**low risk**)	Low risk	Low risk	Some concerns
Zhou 2024	Computer-generated randomization, the sequence was concealed from all investigators (**low risk**)	**Open label trials**, deviations from established intervention measures do not affect objective outcome (*H. pylori* eradication rate), but may affect subjective outcomes (adverse events and compliance)	Although there were some missing data, the study conducted both ITT and PP analyses, and there is evidence that the result was not biased by missing	Low risk	Low risk	Low risk

			outcome data (**low risk**)			
Tai 2025	Computer-generated randomization, Sequentially numbered, opaque, sealed envelopes (**low risk**)	**Open label trials**, deviations from established intervention measures do not affect objective outcome (*H. pylori* eradication rate), but may affect subjective outcomes (adverse events and compliance)	Although there were some missing data, the study conducted both ITT and PP analyses, and there is evidence that the result was not biased by missing outcome data (**low risk**)	Low risk	Low risk	Low risk
